# Rethinking conformity and imitation: divergence, convergence, and social understanding

**DOI:** 10.3389/fpsyg.2014.00726

**Published:** 2014-07-08

**Authors:** Bert H. Hodges

**Affiliations:** ^1^Department of Psychology, Gordon CollegeWenham, MA, USA; ^2^Department of Psychology, University of ConnecticutStorrs, CT, USA

**Keywords:** conformity, dissent, imitation, pragmatics, social learning, trust, truth, understanding

## Abstract

Social and developmental psychologists have stressed the pervasiveness and strength of humans’ tendencies to conform and to imitate, and social anthropologists have argued that these tendencies are crucial to the formation of cultures. Research from four domains is reviewed and elaborated to show that divergence is also pervasive and potent, and it is interwoven with convergence in a complex set of dynamics that is often unnoticed or minimized. First, classic research in social conformity is reinterpreted in terms of truth, trust, and social solidarity, revealing that dissent is its most salient feature. Second, recent studies of children’s use of testimony to guide action reveal a surprisingly sophisticated balance of trust and prudence, and a concern for truth and charity. Third, new experiments indicate that people diverge from others even under conditions where conformity seems assured. Fourth, current studies of imitation provide strong evidence that children are both selective and faithful in who, what, and why they follow others. All of the evidence reviewed points toward children and adults as being engaged, embodied partners with others, motivated to learn and understand the world, others, and themselves in ways that go beyond goals and rules, prediction and control. Even young children act as if they are in a dialogical relationship with others and the world, rather than acting as if they are solo explorers or blind followers. Overall, the evidence supports the hypothesis that social understanding cannot be reduced to convergence or divergence, but includes ongoing activities that seek greater comprehensiveness and complexity in the ability to act and interact effectively, appropriately, and with integrity.

## INTRODUCTION

One of the deepest assumptions of social psychology, and many allied disciplines, is that people have strong tendencies to conform, to imitate, to mimic, and to obey. These tendencies are claimed as the basis for coordination, communication, and culture (e.g., Richerson and Boyd, 2005; Mesoudi, 2009). The available evidence, though, reveals a far more complex and interesting set of dynamics: divergence is as pervasive as convergence, but its appearance is often unnoticed or minimized (Berger and Heath, 2008; Haslam and Reicher, 2012; Reicher et al., 2012; Hodges, in press). The story that needs to be told, though, is not simply that we need to pay more attention to divergence, but that we need to appreciate a larger set of dynamics: social understanding cannot be reduced to convergence or divergence.

Why have researchers and theorists been so slow to recognize the importance of divergence, disagreement, diversity, and dissent in the dynamics of social interaction? There are many reasons, but chief among them are theoretical and methodological biases that focus on what might be called “Cartesian individuals”—isolated individuals, separated from the world and others, thinking about how to achieve egoistic goals (e.g., to be accurate, to belong). From this perspective, others come to be treated as means to individually determined ends, rather than partners who must act together to learn and to care for each other and the larger ecosystems of which they are a part. Cartesian thinkers must either try (1) to infer what other isolated individuals are thinking or (2) to project their own thoughts onto others, and simulate what they might do in their situation. The first possibility is chancy at best; social understanding becomes a guessing game that is prey to the constant worry that one has guessed wrong. The second possibility, which initially inspires more confidence, is based on a crucial assumption that the other is similar to the self. The risk is that the assumption is presumptuous, that it hides real and important differences (Reddy, 2008).

The fascination of social psychologists with conformity and other forms of convergence is a consequence of their having begun their work with assumptions of individualism, independence, and isolation (Shotter, 2001). Given these assumptions, it might seem important to show how people influence each other, form common bonds, and productively pursue common goals. Divergence, in contrast, would be seen as relatively uninteresting, since it is a natural consequence of the independence and isolation of individual thinkers. However, I will argue that social understanding is more about embodied joint activity among people across time and task than it is about one individual generating ideas about another in order to predict and control outcomes. Rather than controlling outcomes, joint activities make participants more vulnerable to others, and more dependent on the environment; nevertheless, it also increases the flexibility and integrity of their actions and choices.

An array of studies will be described indicating that people act less like Cartesian thinkers and more like social, embodied, dialogical partners, working together to learn how to act in ways that are good for themselves and their ecosystems. The evidence suggests that people are motivated to *understand* situations, others, and themselves, not simply to seek predetermined goals. Social understanding, as it will be addressed in this article, is not about trying to make the other like the self, or the self like the other, but is about jointly exploring a more comprehensive and complex field of action than any of its participants could have predicted or imagined alone.

The evidence to be presented comes primarily from studies in developmental and social psychology, but it has implications for studies in anthropology, language, learning, and many other domains. The four topics that will be addressed are (1) reinterpretations of classic studies in conformity that shift the focus to truth, trust, and social solidarity; (2) recent studies of the role of testimony and trust in children’s actions and choices; (3) new experiments showing that people do not always conform, even when it is normatively expected; and (4) studies of imitation showing that children are surprisingly selective and careful in their following the lead of others. The evidence suggests that divergence is as newsworthy as convergence, and that there is much yet to learn about how these dynamics interact and play out in social understanding.

## THREE THEMES AND A HYPOTHESIS

Before the evidence itself is presented, three themes should be noted—social understanding, embodiment, and intersubjectivity. These are provided by the Research Topic (*Towards an embodied science of intersubjectivity: Widening the scope of social understanding research*) to which this article contributes. What I take those terms to mean will become increasingly clear in the ways that I make use of them, but it may help to sketch briefly their potential before delving into the details of divergence and convergence in social interaction.

### SOCIAL UNDERSTANDING

The working hypothesis explored in this paper comes from research applying values-realizing theory to social cognition (Hodges and Geyer, 2006; Hodges et al., 2014), perception–action (e.g., Hodges and Lindhiem, 2006; Hodges, 2007b), language (Hodges, 2007a, 2009), and developmental psychology (Hodges and Baron, 1992; Hodges, in press). Values-realizing theory claims that perception, action, and cognition are motivated by values, including clarity, coherence, comprehensiveness, and complexity (Hodges, 2009). The hypothesis is this: *understanding* is the ongoing activity of seeking comprehensiveness and complexity in our knowing and doing. While the values of clarity and coherence point to the need to differentiate and organize our experiences in meaningful ways, comprehensiveness and complexity pull activity toward larger, differing contexts that lead to continuities and discontinuities with prior experience. In an important sense, understanding enlarges and complicates our views and actions rather than satisfying and simplifying them. More specifically, this hypothesis suggests that social understanding is the ongoing activity of divergence, not just convergence, of opening up new possibilities, not simply closing in on predetermined goals. Different people in different positions at different times interacting on common ground provide the basis for exploration, as well as a surer grasp of “this place and time and our identity in it” than when one person guesses or simulates.

### EMBODIMENT

As Wilson and Golonka (2013, p. 1) have suggested, embodied cognition is not the claim that bodies affect minds, but that skilled (mindful) action is a distributed set of physical relationships over time, “brain, body, and environment, coupled together via our perceptual systems.” The examples to be considered will illustrate how social understanding involves multiple bodies interacting together, and how actions made by any one body are dependent on the presence, placement, and activity of other bodies over time. Following the lead of another person (or not) is more than guessing or projecting. It is a search to find the integrity of relationships, physical and social; it is also a search for social solidarity and truth. If so, how is that search carried out?

### INTERSUBJECTIVITY

As the earlier discussion of Cartesian perspectives on social knowledge suggested, intersubjectivity is usually taken to be the relation among independent, disembodied minds. If, however, it is an embodied social activity that pulls us beyond the common ground on which we stand toward a richer appreciation of the larger environment and the broader community within which we dwell, then we have the beginnings of an alternative approach, what might be called interaction theory (De Jaegher et al., 2010) or dialogical theory (Linell, 2009). That is, the way in which we come to know and understand others and ourselves is through engagement with each other.

Engagement takes the situated community as fundamental. There is no gap between individuals requiring a theory of or a simulation of “other minds,” and there is no dispassionate observation of the world from a distance (Reddy, 2008). Rather, humans interact with each other and their environment in variety of meaningful ways, and in doing so they come to learn what the world is, who others are, and who they themselves are. Perceiving oneself, others, and the world are interwoven activities and may be direct (rather than inferred), but only over time, and in ways that require participants to be committed as embodied, engaged presences (Gallagher, 2008; De Jaegher, 2009; De Jaegher et al., 2010).

A central issue to be addressed in this paper is how the perceptions and actions of others are integrated with one’s own actions and perceptions, and how this is constrained by embodied locations and specific social understandings. This integration is necessary for learning and language to occur, and it emerges from the values that make these activities possible (Hodges, 2009). This interactive, dialogical, engaged way of enacting social understanding has been challenging, both theoretically and empirically, for psychologists and other researchers to address adequately. Nevertheless, even traditional methods of investigation, which focus on particular individuals, have revealed the social, ecological, and dialogical nature of social understanding. Thus, we now turn to how social understanding, embodiment, and intersubjectivity emerge from studies on conformity, imitation, and trust.

## DISSENTING FOR TRUTH

One of the most famous studies in social psychology is Asch’s (1951, 1956) experimental dilemma in which he had confederates answer clear factual questions about lengths of lines incorrectly some of the time. Having heard the same wrong answer multiple times, the real participant was in an awkward position: he could say what he thought was false, or he could dissent from a unanimous majority. Asch’s work is the *locus classicus* for claims about conformity among humans because people agreed with the confederates’ wrong answers about 1/3 of the time, far more often than Asch expected. That is an impressive finding, but even more impressive is how often a lone participant told the truth about what he saw in the face of a unanimous consensus to the contrary. Unfortunately, the former finding has attracted virtually all the attention. Despite being the most cited reference to support the power of conformity, Asch’s experiments are a powerful testament to divergence (Hodges and Geyer, 2006). Participants disagreed 2/3 of the time with a 100% consensus, and 95% of the time with a consensus over 80% (Asch, 1956).

What prompted this stunning display of dissent? The simple answer is truth-telling: it was when the majority answered incorrectly that participants disagreed a large majority of the time. How did a story of divergence turn into one of convergence? One crucial reason is that the experiments are not framed in terms of pragmatic actions, multiple relationships, and temporal dynamics, but rather in terms of an isolated Cartesian knower guessing and worrying. One explanation of Asch’s (1956) results assumes that individuals in the experiment are confused by the misleading information and are unsure what is correct, so they *guess* it best to follow the lead of others. A second explanation claims that people realize what the correct answer is, but *worry* that if they disagree with others, they will be ostracized or embarrassed in some way; thus, in order to be liked by others, they agree with their wrong answers (Campbell and Fairey, 1989). Neither of these explanations actually explains the data.

These accounts do not even try to explain all the data, but focus only on incorrect, agreeing answers. This is startling on three counts. First, they take accuracy and dissent to be obvious and psychologically uninteresting. Second, they ignore the diversity of responses to the situation, which ranged from never conforming (26%) to conforming a majority of the time (28%). Third, they completely overlook the most obvious group to describe, the typical participants (the middle 46%), who dissent nine times and agree three times (i.e., the median) on critical trials (Hodges and Geyer, 2006). If Asch’s participants were worried about being liked or being correct, why would they have disagreed so often, or agreed so little?

### ENGAGEMENT, EMBODIMENT, AND UNDERSTANDING

Hodges and Geyer (2006) proposed a new approach to understanding the Asch dilemma that attempts to address weaknesses of earlier interpretations. First, they suggested that Asch’s participants were mostly neither the cowardly conformists that many social psychologists have portrayed, nor the independent truth-tellers Asch was looking for; rather, they were ecologically sensitive, pragmatically astute individuals who were trying to be truthful and cooperative in a complex and awkward situation.

Second, Hodges and Geyer (2006) argued that there are multiple values—truth, trust, and social solidarity—that properly constrain Asch’s participants. As Asch (1990) realized, truth matters to people, and he chided his social psychological colleagues for not acknowledging this fundamental fact. On the other hand, he saw the social influence of consensus as a danger (Asch, 1952). Despite Asch’s reservations, trusting others and expressing social solidarity are not wrong: without them, the recognition and expression of truth itself would be hampered (Campbell, 1990). Asch’s situation is not a simple choice between good and bad, between truth-telling and cowardice, but a delicate task of coordination: how can a participant speak truthfully in a way that honors his/her own view, that respects the views of others, and that answers appropriately to the experimenter?

Third, to pull off this coordination Hodges and Geyer (2006) hypothesized that many participants are engaging others in a nuanced, respectful way, varying their answers over trials rather than being trapped by an all-or-nothing choice. The 9/3 disagree/agree pattern of typical participants indicates clearly and truthfully their dissent from the consensus, yet it also respectfully acknowledges that consensus by repeating it occasionally. Hodges and Geyer (2006) claim that participants make local errors in an attempt to express a larger truth; that is, that they are in an awkward, frustrating situation in which there are tensions among multiple obligations. Although participants appear to be inconsistent, it is more likely that they are working to realize multiple values that are in tension. Almost certainly this is not a conscious strategy, but rather a product of a continuously evolving dynamical system in which prior choices constrain current ones (cf., Thelen et al., 2001).

Fourth, intersubjectivity appears in the Asch (1956) studies in the pragmatics of the quasi-conversation that the experiment is. Hodges and Geyer (2006) suggest that Asch’s account is not sufficiently sensitive to participants’ multiple obligations and to the conversational nature of the interaction among the real participant, their peers, and the experimenter. If a person always dissented from a group’s expressed views (i.e., what Asch hoped would happen), it would be easy for that person to be seen as arrogant or dismissive. If, on the other hand, one agrees some of the time with incorrect answers, it functions as a pragmatic signal of one’s commitment to taking others’ views seriously (i.e., social solidarity) and one’s openness to further engagement (i.e., trust) in the strange situation in which they find themselves.

Fifth, social solidarity must be maintained if one is to be taken as a serious witness to the truth of matters. If there is a lack of trust between parties, truth telling becomes much more delicate and difficult. Dissent cannot function if it is directed toward people who do not care what others think, or if there is no concern for those to whom the dissent is addressed. Dissent implicitly appeals to some sense of shared concern for truth and other values that provide the common ground for communicative discourse and social interaction.

Sixth, regarding embodiment, there is some evidence that it matters that participants are physically present and confronting each other as well as the experimenter. Attempts that soon followed Asch (1951) tried to isolate the participant in a literal Cartesian room to see how virtual group members (simulated by the experimenter) would create the social pressure that was assumed to produce Asch’s results. The Crutchfield (1955) procedure generally yields less agreement with wrong answers (Bond and Smith, 1996). This suggests that the physical–moral presence of others who speak to the participant, and the participant to them, contributes to the nature of the dilemma itself, as well as to the common ground necessary to address it.

To summarize, Asch’s (1956) participants were not simply facing an epistemic quandary, about which they might guess or worry. Rather they were in a social-moral dilemma: what does one say in a frustrating situation, when one is facing two bad choices, either to speak truthfully and forcefully, but in a way that risks being perceived as disrespectful, or to speak with greater tact and humility, but at the risk of denying one’s own convictions. Both of these options were chosen, but far more often Asch’s participants varied dissents and agreements over time. Thus, dynamics of divergence and convergence were intertwined, revealing an embodied engagement with others that worked to honor truth, while also being sensitive to multiple relationships and multiple obligations. The hope of participants seems to be that if they say what they see, but also take account of others, perhaps together they can learn what kind of situation they are in and what to make of their disagreement. Engagement and dialogical interaction seek social understanding (i.e., a larger, richer appreciation of oneself, others, and the setting), rather than simply predicting or projecting.

## TRUST AND GUIDANCE

If there is any place where we expect to find widespread tendencies to follow the lead of others and to conform to observed practices, it is among children. What patterns of convergence and divergence have emerged in studies of social development? Young children—widely believed to be gullible conformists by some, and independent investigators by others—show surprising sophistication in terms of evaluating the worth of others’ testimony about events in the world (Kuczynski and Hildebrandt, 1997; Harris, 2012). As is true of adults, children take account of their own perceptual experience of events and possibilities, but they also are guided by the perceptions and actions of others. Their actions and choices suggest they have social understanding, founded in embodied interactivity with others over time.

Recent research indicates that children’s epistemic judgments reveal both more vigilance (Sperber et al., 2010) and more trust (Harris, 2012) than developmental psychologists generally have been willing to grant. For example, children trust those who have shown themselves reliable in the past, but they are not indiscriminate in that trust. If the more reliable informant is in a bad position to see the relevant information, children tend to trust a less reliable but better positioned informant (Corriveau and Harris, 2009; Brosseau-Liard and Birch, 2011). They even seem to operate on a principle of charity: they are willing to learn from an informant who had previously been incorrect, if the informant’s position had prevented him or her from seeing the relevant information. However, they discount information from someone who previously had been in a good position but was inaccurate (Nurmsoo and Robinson, 2009).

Children tend to choose other children to learn the affordances of novel toys, but they prefer adults as the best sources for names of new objects. In short, they respect the relevance of interactivity: they prefer to use guides more likely to have had relevant experience (e.g., VanderBorght and Jaswal, 2009; Rakoczy et al., 2010; Sobel and Corriveau, 2010; Koenig and Jaswal, 2011). They show a preference for first-hand testimony over second-hand evidence (Einav and Robinson, 2011), and they also show a preference for information that is consensually agreed upon by several adult witnesses, compared to a dissenter’s claim (Corriveau et al., 2009). However, if an adult makes a claim that contradicts the child’s own direct experience, children tend to question or correct the adult, rather than accepting the adult’s mistaken claim (Koenig and Echols, 2003). If multiple adults make false statements (e.g., about the color of toy), most children state the correct color, but a minority follows the lead of the adults (Clément et al., 2004).

Two recent studies worked out versions of an Asch (1951) dilemma to present to 3 to 4-year-old children, one with a consensus of peers (Haun and Tomasello, 2011), and one with a consensus of adults (Corriveau and Harris, 2010). Their most stunning finding was how often children dissented from unanimous majorities: for example, 76% of 4 year olds and 58% of 3 year olds answered correctly every time in Corriveau and Harris (2010). In this same study, children increasingly dissented from incorrect majorities over succeeding answers, and when some clear, relevant good was at stake, they never agreed with incorrect adults (i.e., the child could win a prize, if they picked a bridge of the right length to cross a river in a game). This is dramatic evidence that children trust their own eyes, and are willing to disagree with a consensus of adults who answer incorrectly. On the other hand, they also show sensitivity to social consensus, at least when decisions do not appear particularly consequential.

One feature, related to embodiment is noteworthy. Corriveau and Harris used videotaped adults as their majority. Haun and Tomasello (2011) believed that stronger evidence of conformity in children could be found if there was face-to-face contact, and if the others involved were age-peers, not adults. They devised a procedure with four children, each looking at a book that presumably was the same for all; however, one child’s book differed on selected pages. They found somewhat more conformity than Corriveau and Harris did (about 34%), but otherwise the picture that emerges is almost identical. Haun and Tomasello refer to the willingness to say things publicly that one does not find personally convincing “strong conformity” and they claim their experiments show children do this. However, the studies provide far more compelling evidence for children’s clarity and conviction. Children, like adults, appear to be truth-tellers who are sensitive both to the information value of others’ claims and to the pragmatic complexities of dissent and agreement with others.

Other evidence from developmental studies also yields the same pattern of cooperative engagement with others, but a strong tendency of children to trust their own perception-action capabilities. For example, when children are deciding whether to step across a gap in their surface of support that is sufficiently wide and deep that they hesitate, they often look to a parent for clarification, to see if they are smiling, frowning, or looking uncertain. What happens when the child perceives that the gap is crossable, but the parent discourages the action? Individual differences are considerable, but most children take the step, as if they were saying, “Mother knows best, but sometimes I know better” (Feinman, 1992, p. 252). Other studies using multiple sources of information have found that children generally look to knowledgeable sources more than attractive ones to clarify the situation. Children confronted by an unexplained object in the room look more readily at a stranger who appears confident about the object’s meaning rather than looking at a more familiar and attractive person (e.g., their mother) who appears puzzled (Feinman, 1992).

Research on children’s reactions to parental commands and instructions (Kuczynski and Kochanska, 1990; Kuczynski and Hildebrandt, 1997) indicates that children generally are cooperative, but they also engage in a number of actions that exhibit their own agency (e.g., complaining, arguing, partial compliance). Matas et al. (1978, p. 554) argued that “the competent 2-year-old … is not the child who automatically complies … when requested to stop playing and clean up the toys, but who gradually cooperates with the mother.” Overall, children care about truth, not just approval, and engage in more dissent than is generally appreciated. Furthermore, their concern for truth and dissent is not so much a denial of their involvement in social relationships, as it is a sign of their commitment to them (Kuczynski and Hildebrandt, 1997). Reddy (1991, p. 144) provides evidence that this paradox of commitment and divergence begins prior to the end of the first year, when children initiate opposition to caretakers’ actions and directives in a manner that can only be described as teasing. She observes that teasing is not so much a particular pattern of action but “is an element in a relationship,” one that can bring its members closer together.

Overall, the picture that emerges from studies of children’s trust in and use of testimony and advice from others suggests a developing sophistication that is surprisingly comprehensive and complex. Mostly children pay attention to embodied interactions of others and their likelihood of having observed or encountered relevant information. They do not seem to be guessing or projecting primarily, but interacting and acting in ways that are engaged, trusting, and vigilant. Even young children have a remarkably subtle understanding of relationships, timing, location, and how to find integrity. For the most part, children appear to act as dialogical partners, rather than blind followers or solo explorers.

## SPEAKING FROM IGNORANCE

It is often assumed that children are in a position of ignorance, in need of guidance from adults and older children to direct their efforts. As the research just reviewed indicates, children seem to share that conviction, but they also show a surprising confidence in their own abilities to see and know, and considerable flexibility in how they integrate their own perspectives with those of various others. Acting from ignorance, however, is not confined to children. Adults are learners too, and they often find themselves in a position of ignorance with respect to others who know more. Do they trust and follow others’ lead, or do they ignore others and follow their own counsel?

Hodges et al. (2014) explored this question by placing people in different positions relative to a screen so that two (A and B) could see information clearly, and one (C) could not. Furthermore, participants at C could easily see that A and B were better positioned than they were. They were then asked about information projected on the screen (e.g., superimposed words embedded in patterns). On critical trials participants at C had no definitive information with which to answer independently (e.g., they could see isolated letters but not the particular word about which they were questioned). However, they heard two other people (A and B) confidently give the correct answer before it was their turn.

Asch was surprised that people ever agreed with others’ wrong answers. In contrast, the Hodges et al. (2014) experiment inverts the Asch situation: agreeing with others’ answers appears to be the only sensible thing to do. However, Hodges et al. (2014) predicted that participants would surprisingly often violate this expectation: they would make up their own, incorrect answers rather than repeating the correct answer given by A and B. This disagreeing with wrong answers, which they called the speaking-from-ignorance (SFI) effect, occurred about 30% of the time in several experiments. Further evidence indicated that participants were knowingly choosing not to agree with answers they believed were correct.

This result seems quite implausible at first. Unlike the Asch situation where there is a contradiction between perspectives, there is no contradiction in the SFI situation; thus, it seems there should be no dilemma. However, Hodges et al. (2014) found that participants do experience the situation as a dilemma. The reasons they do can be framed in terms of intersubjective engagement and embodiment. If the SFI situation, like the Asch situation is seen as a sort of conversation, then pragmatic constraints come into play. Pragmatic cooperativeness usually entails saying neither what you believe to be false, nor that for which you lack adequate evidence (Grice, 1975). However, an SFI situation pulls and twists these two aspects of cooperation inside out, creating a frustrating tension. While it is perfectly possible and appropriate to repeat what other, better-informed people have told you—it seems a simple matter of trust—many participants feel it is not quite right. “It feels like it’s cheating,” is the way some expressed it. The embodied location of each of the participants and the timing of their answers matters, and many participants feel a sense of obligation to be true to their position, as well as to the timing of their answer. Answering last affords them the option of answering correctly with considerable confidence, and about 50% of all participants always do so. However, their embodied position makes this awkward. The SFI effect reveals an understanding of the situation that is truthful and pragmatic: I cannot see from my position, so it is difficult for me to answer correctly and to do so with pragmatic warrant. This understanding of the situation, both in terms of dialogical relationships and in terms of embodied locations, constrains many participants to go beyond immediate tendencies to “be correct” or “be agreeable.”

Hodges et al. (2014) propose that the same dynamics at work in the Asch situation are also at work in the SFI situation—truth, social solidarity, and trust. Answering incorrectly, and disagreeing with better informed others, may seem irrational, but doing so truthfully acknowledges one’s ignorance, concretely expressing one’s commitment to truthfulness, not simply to being correct. It is also an expression of vulnerability and therefore it indicates trust in others’ ability and willingness to appreciate the awkwardness of one’s position and to continue to share their knowledge. Although social solidarity generally leads toward agreement, it goes beyond uniformity and consensus: it encourages each participant in a group to make his or her unique contribution to the integrity and well being of the group as a whole. Thus, at the level of conversational pragmatics, social solidarity leads each participant to want to make a distinctive contribution to the conversation, rather than blindly repeating what others have said. It is not wrong, of course, to repeat others when one is in a position of ignorance. For example, we generally expect students to repeat what their teachers tell them. However, we also expect students to offer their own answers, even when those answers are awkward or incorrect, an every day exemplar of an SFI effect.

To test the hypothesis that pragmatic constraints to speak truthfully and with epistemic warrant lead participants to disagree with correct answers sometimes, Hodges et al. (2014, Experiment 3) compared groups, one of which was primed to be particularly sensitive to the demands of honesty. Even though participants were given the opportunity of winning a monetary prize by answering correctly, 49% of the time participants in the honesty-prime condition chose not to agree with correct answers given by others, compared to 19% in the no-prime condition. Along with other findings of other experiments, the results suggest that observed incorrect, non-agreeing answers were “not a speaking-last effect, a speaking-from-a-different-position effect, a speaking-to-differentiate [oneself from others] effect, or a self-presentation effect (e.g., drawing attention to oneself as unique or creative)” (Hodges et al., 2014, p. 228). Rather, it is a speaking-from-ignorance effect that is yielded by the dynamics of truth, trust, and social solidarity.

Engagement in the SFI situation requires attending to embodied selves. Participants can see others are better positioned than they themselves are, yet they do not always agree because they sense a responsibility to their own physical, social, and moral location in the experimental setup. Answers reflect the layout of the situation as a whole, and the interdependence among positions, not simply a choice of one perspective or another. Even when participants gave agreeing answers, which they did most of the time, many participants exhibited (as informally observed by the author) bodily tension when they were giving correct, agreeing answers (e.g., they lowered their voice as if embarrassed, they jiggled their pencil, they hesitated, they tried to sound like they were saying something novel rather than repeating others). Most likely, this tension emerged because they were aware that their position both did and did not warrant their correctness.

To appreciate how social understanding is operative in the SFI effect, one needs to think of social learning at the communal and historical levels. What is necessary for cultures to function effectively in terms of learning and sharing knowledge? Much attention has been paid of late to the importance of agreement, conformity, and faithful replication in the constituting of cultures (Richerson and Boyd, 2005; Mesoudi, 2009). However, there is also a need for innovation, creativity, and the ability to share and elaborate those discoveries. Cultures necessarily embody a tension between sharing common practices (i.e., homogeneity) and the production of new variations (i.e., heterogeneity) from which better tools and skills can emerge (Hodges, in press).

The SFI experiments suggest that it is better if not everyone agrees with expert opinion or the consensus judgment, at least all the time. The general wisdom embodied in this tendency is that it may be better not to follow others blindly, even if they seem to be in the position of the expert. Scientists are often annoyed when others do not follow their lead, but there is good reason for people to be cautious. People know things are more complicated than even experts can appreciate, and they know that science itself depends on people willing to challenge the consensus and to propose ideas that may seem crazy or impossible, at least at first. In any event the SFI effect shows that people’s use of others’ testimony is not simply a goal-driven, rule-following activity, but engages the dynamic interplay of divergence and convergence to realize values that may be more complex and further afield than answering the next question correctly.

## SELECTIVE, FAITHFUL IMITATION

Imitation, “matching the behavior of a model after observing it” (Over and Carpenter, 2012, p. 183), is a kind of conformity, although it is rarely treated as such. The main difference is whether a group or an individual is being imitated. One of the most basic facts of imitation, although often overlooked, is that it is selective: who and what is copied, when and how, are basic questions. Behind these questions is a still deeper one: why does imitation occur?

### WHAT IS IMITATED AND HOW?

Despite the intentional character of imitation, Horowitz (2003) has argued that what counts as imitation is vague. She studied chimpanzees and adult humans and found that both tended to copy a complex series of actions partially. Both noticed easier ways to solve the puzzle she presented, so that even adult humans who explicitly claimed to be imitating exactly failed to do so. A crucial issue is that it is experimenters who decide what is to count as relevant to the action to be imitated. Must the one imitating use the same hand as the model, use the yellow ball rather than blue ball, and so on, for it to be counted as matching the model? The relevance question is, of course, one of the most challenging in psychology. Deciding what is relevant demands a larger context of history, function, and purpose; it raises the question of why imitation exists and what it does in the larger scheme of things. One of the most active discussions among researchers in this regard is whether imitation is primarily a way of learning from others about the world, or whether its focus is more on developing relationships (i.e., identifying or communicating with the model; Over and Carpenter, 2013).

Tomasello (1999) claimed that children imitate much more faithfully than chimpanzees, and subsequent work has substantiated that children are far more likely to copy causally irrelevant actions performed by an adult model in solving a puzzle (e.g., getting a piece of food) than chimpanzees who choose more efficient means of solving the puzzle (Whiten et al., 2009). While this has led some social anthropologists to refer to children’s close copying as *over-imitation* (Lyons et al., 2007), implying that it is excessive or “blinkered” (Whiten et al., 2009, p. 2425), others have taken a far more positive view of the tendency, considering it *faithful* or high fidelity imitation (Nielsen and Blank, 2011; Over and Carpenter, 2012). The latter have seen it as contributing to the human propensity to transmit cultural patterns faithfully, allowing those patterns to spread and survive (Richerson and Boyd, 2005; Nielsen and Tomaselli, 2010).

How are children’s imitative actions both selective and faithful? How and why do children sometimes imitate quite precisely and other times much more selectively? These are central questions now being addressed by researchers, and how they should be answered are matters of ongoing discussion and debate (Nielsen and Blank, 2011; Over and Carpenter, 2012)? I will not try to resolve all the difficulties, but it is interesting that imitation researchers are now appealing to social psychology and its views of conformity and mimicry to argue their cases (e.g., Over and Carpenter, 2013). Perhaps, the more complex views of trust and prudence, of agreement and dissent, discussed earlier can provide fresh perspectives on imitation as well.

The possibility explored in this section is that children’s imitative acts are seeking understanding, rather than simply being acts of learning or acts of affiliation. I will argue that imitative actions are selective and faithful, not one or the other, but they also go beyond what these two terms suggest. A powerful exemplar of this claim is that children tend to copy intentional actions of others, but not others’ mistakes or their failed attempts. If adult models begin but do not complete an action (e.g., pulling a top off), children tend to complete the action they saw partially done (Meltzoff, 1995; Nielsen, 2009). If they hear a puppet make a mistake in saying a sentence, repeating a word that is unnecessary, they tend to omit the word when they repeat the sentence (Over and Gattis, 2010). Children’s perception of agency appears to be crucial: if the action is “modeled” by a machine or an inanimate toy, they imitate its movements more literally and less often. This replication of intention rather than repetition of observed action, which begins in the first year (Nielsen, 2009), indicates that what is being matched is ecological and prospective. It suggests that what motivates imitation is larger than simply learning about things, or simply affiliating with the model who has served as demonstrator.

### WHEN DOES IMITATION OCCUR?

There are a variety of conditions that affect the selectivity and faithfulness of imitative precision and completeness. One is the transparency of intentions. If an adult turns on a light switch with her head instead of her hands, children will imitate her action, but only if the adult’s hands are empty. If the adult’s hands are occupied, then the children imitate turning on the light, but they do it with their hands (Gergely et al., 2002), illustrating both selectivity and faithfulness. More generally, children tend to imitate less faithfully in tasks that have a clear goal (e.g., extracting a prize from a puzzle box): they tend to omit extra motions and actions that do not contribute directly to extracting the prize (Horner and Whiten, 2005; Kenward et al., 2011). However, if the causal mechanisms of the puzzle are opaque, then the model’s movements are followed more closely (e.g., Lyons et al., 2007). Thus, a second condition constraining selectivity is the transparency of the goal and the means of its achievement. A third trend is the increasing faithfulness of replication as children become older. In fact, adults sometimes imitate more completely and accurately than children: with no instructions to imitate adults imitated more than 5 and 3 year olds, and the older children included more causally irrelevant actions than the younger ones did (McGuigan et al., 2011). Fourth, children who are uncertain about how to solve a problem, or who have tried previously and failed at a task, tend to copy a model’s actions much more faithfully than if they have not had difficulty (Williamson et al., 2008). Fifth, children who have been primed with social exclusion tend to imitate models more closely (Over and Carpenter, 2009).

Finally, there are two other situations that tend to yield more faithful imitation by children. One is when adults signal that they are intending to teach the child (Brugger et al., 2007; Bonawitz et al., 2011), and the other is when models demonstrate competence rather than ineptness (DiYanni and Kelemen, 2008). If children see an adult demonstrate a puzzle solution several times, they tend to imitate the demonstrator’s actions, even if those actions do not appear to be necessary, but only when that particular demonstrator is present (Nielsen and Blank, 2011). This tendency of the child to take into account a demonstrator’s particular way of achieving an outcome, rather than simply taking the shortest, most direct route to an outcome, is one that Nielsen and Blank argue is important for the development of cultural groups, including their diversity and richness. Nielsen and Tomaselli (2010) suggest that this tendency to attend to particular cultural ways of doing tasks appears in all kinds of cultures, and leads children (and later, adults) to engage in actions that may interfere with what they as individuals might desire or believe. They claim that this tendency to follow others’ lead is neither blind nor maladaptive. Rather, it is a mark of humans’ tendency to trust others to alert them to complexities of physical causality that are not easily observed, as well as helping to enhance social solidarity with other members of their culture.

These last two factors affecting faithfulness lead us to notice more carefully the question of who the model is in relation to the child. Are some models imitated more than others?

### WHO IS IMITATED?

Human infants and children tend to choose as models those who have imitated them (Over et al., 2013), who are warm and friendly (Nielsen, 2006), who have acted reliably in the past (Clément et al., 2004), and who are ingroup members (i.e., use child’s native language rather than another; Kinzler et al., 2011). Perhaps, the broadest pattern that emerges is that embodied engagement, and dialogical interactivity leads to greater imitation. Children are more likely to imitate faithfully if there is intersubjective engagement of adult and child prior to the demonstration that will serve as the test of imitation. Imitation is increased if the adult plays with the child prior to the test, or talks with them, and if the child is particularly tuned to interacting with others (Nielsen, 2006; Brugger et al., 2007; Hillbrink et al., 2013). In fact, one way a child and an adult can interact with each other is imitating together (Nielsen et al., 2013).

The increased imitation is tied to the specific individual who has engaged and interacted with the child previously or in the larger context in which the imitation task per se is embedded (Yu and Kushnir, 2014). Embodiment, as well as specificity, matters: imitation occurs markedly less when videotaped demonstrators are presented rather than live demonstrators (McGuigan et al., 2007; Nielsen et al., 2008). Thus, intersubjective engagement seems to encourage imitative behavior, and it is not due to some general increase in arousal, attention, or receptivity. The engagement is dialogical, concerted, and embodied: children imitate *with* others, not simply as a response to an action or a movement, but as a dialogical activity with a particular other person with whom they are engaged socially and physically.

The large-scale picture that emerges from these studies is that children do not simply converge with those whom they observe, nor do they diverge as if alienated. Children have a natural affinity for convergence, but not with just anyone, or anything, or under any circumstance. They seem to be attuned to others that care about them, and to those situations in which there is something to learn and something to share.

### WHY DOES IMITATION OCCUR?

One possibility, still widely taken for granted, is that imitation in infants and young children is some hard-wired tendency to repeat what they observe, and should not be taken as intentional action (Lyons et al., 2007). All the evidence reviewed above suggests otherwise. Imitation is far too selective and varies too much in its fidelity to be some form of automatic motor mimicry (if such a thing exists at all). Over and Carpenter (2012) proposed that imitation is motivated in three ways. First, children are motivated to learn about the world, and to use others to do so. Second, they are also motivated to identify with the person being imitated and the larger social activities they embody. Third, children are sensitive to social pressures that encourage particular ways of doing things. It is the latter two conditions, they propose, that encourage more specific, detailed, and complete copying. Finally, they claim that no existing theory of imitation does a good job of accounting for existing evidence along these three motivational axes.

The challenges to imitation researchers go even deeper, though, than Over and Carpenter’s (2012) critique. Consider, for example, two recent experiments. Buttelmann et al. (2013), as well as Yu and Kushnir (2014), find a substantial number of children, sometimes a majority, who do not choose to follow either of two models that are presented, or who engage in an action other than the two options in which the experimenter was interested. For example, 14-month-old children watch a model, who has previously spoken either German (the child’s language) or Russian to them, turn a light on with his head. There is more imitation of the German speaker’s action, but an even more interesting finding is that a majority of children do not imitate either speaker, but turn the light on in their own way, usually with their hands. When presented with a model that chose one of two objects and acted pleased with his choice, children later showed no preference for the model’s choice in making their own choice. These results seem similar to the frequent finding in social anthropology and psychology that people tend to trust their own judgments and experience (Eriksson and Coultas, 2009; Eriksson and Strimling, 2009; Hodges, in press), and do not follow too readily the lead of others. The irony is that it is procedures and choices of just the sort these two studies consider that are assumed to be most vulnerable to conformity effects.

### SEEKING UNDERSTANDING IN IMITATION

Bråten (2000), Nagy (2006), and Reddy (2008) outline a larger context for understanding imitation, suggesting that it is a primitive dialog, not simply a conduit for passing on expertise, as cultural anthropologists often treat it. Infants initiate actions in an apparent attempt to provoke parents into reacting. These provocations are marked by heart deceleration (symptomatic of anticipation), unlike imitative responses, which show heart acceleration (Nagy and Molnar, 2004). The child and the adult see the other as caring what the other does, and as being open to what the other has to offer. Infants are sensitive to whether others are looking at them or away, and prefer direct visual engagement (Farroni et al., 2002; Rigato et al., 2011). Adult and child have to sense an openness and obligation to each other that is emotional, that indicates “I take you the way you are” and that anticipates what the other might do next (Bråten, 2009). It is this promise of learning together that encourages people to conform to parents, teachers, and colleagues, as well as to challenge and test them in a dialog that appears to begin even before children can speak (Meltzoff and Williamson, 2010).

There is a newfound appreciation among imitation researchers for its social nature (e.g., Over and Carpenter, 2013). However, it appears that they have slipped into the same sorts of dichotomies that befuddle standard explanations of conformity in social psychology (Hodges et al., 2014). One explanation given for faithful imitation in children is that they are predisposed to see any purposeful action by adults as causally relevant (Lyons et al., 2007; Whiten et al., 2009). Another explanation is that faithful imitation arises from children’s increasing sensitivity to cultural norms and their desire to learn the socially approved way to do things (Kenward, 2012). The former is similar to what Deutsch and Gerard (1955) called informational influence (i.e., we conform in order to be correct), and the latter is similar to normative influence (i.e., we conform in order to belong and be liked). However, as was true in the case of conformity, this dichotomy cannot capture the subtlety and sophistication of children’s selective faithfulness.

It appears that children are trying to be faithful to more than norms or causes. Bannard et al. (2013) claim that children act in ways that are precocious, as if they can do more and know more than they are able to achieve and complete. Perhaps, imitation by children is not simply about copying what exists, but more about trying to explore what is promising in the actions of other people and in the events of their environment. The intentional activities of children appear to be exploring something larger and more complex than physical causality and social identity. Hillbrink et al. (2013) suggest that we should look at imitation, not just as an instrumental act, but also as a communicative act that involves reflection on the significance and values of others. If, however, values are not personal and social preferences, but are rather the “global constraints on an ecosystem” (Hodges and Baron, 1992; Hodges, 2007a, 2009), it may be that imitation is a precocious search for the integrity of those ecosystems as a whole.

## CONCLUSION: UNDERSTANDING, DIALOG, AND SURPRISE

All of the phenomena explored in this paper—the Asch effect, social reference effects (i.e., children seeking and using information from others), the speaking from ignorance effect, and imitation effects—yield the same fascinating and deep pattern. People seek and respond in ways that show their propensity for truthful information, for effective action, for social appropriateness, and for trust and prudence.

The evidence from all these domains suggests that people, including children, participate in social–physical encounters as engaged partners, intending to learn about the world, about others, and about themselves in a way that allows them to act appropriately and effectively. In these encounters people pay close attention to the embodied location of themselves and others in judging the worth of testimony by others and in deciding what they themselves should say and do. Furthermore, they show considerable sensitivity to historical patterns: people who have indicated their interest and care previously, and who have provided accurate and useful guidance in the past, are accorded greater deference than those who have been less caring and accurate, or are unknown. Overall, adults and children show considerable sophistication in their ability to integrate information from a variety of sources over time in ways that are appropriate to their immediate physical and social well-being, but that also gives promise of their being able to continue to learn about their social and physical locations and obligations.

The larger picture that emerges is that people are less concerned about predicting and controlling than they are in understanding the world, others, themselves, and how they all fit together. The evidence that has been reviewed suggests that people’s actions reveal that they are seeking something more comprehensive and complex than most theories of conformity and imitation can countenance. Much of the burden of this article has been to show that divergence is far more common and powerful than social and developmental psychologists have acknowledged. When truth is on the line, adults and children defend it against majorities and models that would lead them astray. Nevertheless, across all these domains children and adults show themselves to be sensitive to the worth of others’ perspectives and the need to acknowledge that worth. In the sharpest dilemmas, the diversity of action and choice is considerable, but it indicates that people generally work to find some accommodation that maintains social, physical, and moral integrity.

Finally, children and adults rarely act as if they are Cartesian thinkers, trying to figure out the world on their own. They show ample evidence of being guided by others, but they show a limited appetite for following others blindly or completely. Rather than being independent learners or conformist imitators, they act selectively and prudently to be faithful to the world, to their own perceptions and actions in it, as well as to the perceptions and actions of others. They seem to be looking for a larger, richer understanding that holds these together.

This search can be characterized as a dialog, a conversation among self, others, and the world. Theory and research on conversations and dialog have tended to emphasize alignment: speakers converge on vocabulary, pronunciation, syntax, and many other aspects of language as they talk together. This has led to claims that alignment is necessary to be able to predict what others’ will say and to control one’s own replies (Pickering and Garrod, 2013). This is the same impulse that has allowed psychologists to minimize divergence and selectivity in conformity and imitation. Fusaroli et al. (2012) observed that people who are conversing engage in selective alignment; in fact, they noted that indiscriminate alignment undermined effective performance on the task. Although, it is rarely noted, speakers diverge as much as converge when it comes to what they say and how they say it, varying on virtually every dimension measured by linguists (Strigul, 2009). Perhaps, the most profound fact about dialog is that “it is the things that we cannot predict that are the most important parts of conversation. Otherwise, it is hard to see why we should speak at all” (Howes et al., 2013, p. 359). It is the larger, richer dialog of convergence and divergence that is needed for language, learning, and life to continue. Perhaps, what is most needed for researchers and theorists is to be surprised once again by the dynamics of this dialog.

## Conflict of Interest Statement

The author declares that the research was conducted in the absence of any commercial or financial relationships that could be construed as a potential conflict of interest.
